# Impact of 3-year changes in fasting insulin and insulin resistance indices on incident hypertension: Tehran lipid and glucose study

**DOI:** 10.1186/s12986-019-0402-3

**Published:** 2019-11-09

**Authors:** Aidin Baghbani-Oskouei, Maryam Tohidi, Mitra Hasheminia, Fereidoun Azizi, Farzad Hadaegh

**Affiliations:** 1 Prevention of Metabolic Disorders Research Center, Research Institute for Endocrine Sciences, Shahid Beheshti University of Medical Sciences, Tehran, Iran; 2 Endocrine Research Center, Research Institute for Endocrine Sciences, Shahid Beheshti University of Medical Sciences, Tehran, Iran

**Keywords:** Insulin resistance, HOMA-IR, Insulin-glucose ratio, Hypertension, Change, Cohort study

## Abstract

**Background:**

To examine the association between changes in fasting insulin, homeostasis model assessment of insulin resistance (HOMA-IR), and insulin-glucose ratio (IGR) levels, over approximately 3 years with incident hypertension.

**Methods:**

A total of 2814 Iranian participants (1123 men) without hypertension and known diabetes at baseline and the first examination were followed for a median of 6.32 years. The associations between quartiles of changes in fasting insulin and IR indices with incident hypertension were assessed using multivariate Cox proportional hazard regression analyses with first quartile as reference. The models were adjusted for baseline values of insulin or each IR index, and age, sex, smoking, physical activity, educational levels, marital status, history of cardiovascular diseases, baseline levels of systolic and diastolic blood pressures, estimated glomerular filtration rate, triglycerides, total cholesterol, high-density lipoprotein cholesterol, fasting plasma glucose (only for insulin change) and both body mass index (BMI) per se, and its change. Akaike’s information criteria (AIC) was applied as indicator for goodness of fit of each predictive model. The discrimination ability of models was calculated using the Harrell’s C statistic.

**Results:**

During the study, 594 incident cases of hypertension (253 men) were identified. The 4th quartile of changes in insulin, HOMA-IR, and IGR showed hazard ratios (95% confidence interval) of 1.31 (1.01–1.69), 1.18 (0.92–1.52), and 1.53 (1.18–1.98) for hypertension, respectively, in fully-adjusted models. Changes in fasting insulin levels and IR indices showed significant increasing trends for incident hypertension, moving from 1st to 4th quartiles (all *P*-values < 0.05). Focusing on model fitness, no superiority was found between changes in fasting insulin, HOMA-IR, and IGR to predict incident hypertension. The discriminatory powers of changes in fasting insulin and IR indices as assessed by C index were similar (i.e. about 80%).

**Conclusion:**

Changes in fasting insulin and IR indices were significantly associated with developing hypertension among normotensive population even after considering BMI changes.

## Introduction

Hypertension as a leading cause of cardiovascular morbidity and mortality, remains a persistent public health challenge due to its high prevalence rate worldwide [1]. The total burden of the disease is expected to rise to 1.56 billion by 2025 [1]. Age, sex, body mass index (BMI), diabetes status, and blood pressure variables were reported as the most common predictors for subsequent hypertension in a systematic review on hypertension predicting models [2].

Insulin resistance (IR) and compensatory hyperinsulinemia, as main components of metabolic syndrome, may have serious consequences, contributing to the development of hypertension [3, 4], potentially through adverse effects on adrenal medullary activity and the renin angiotensin system, sympathetic nerve system, and vascular smooth muscle tone [4, 5]. Several methods have been proposed for assessment of IR in humans; among these, the homeostasis model assessment of insulin resistance (HOMA-IR) index is the most well-known [6].

Numerous longitudinal studies have discussed the role of both IR and compensatory hyperinsulinemia in the occurrence of hypertension [7–15]. Wang et al., in a meta-analysis study, showed elevated fasting insulin concentrations and IR were independently correlated with an exacerbated risk of hypertension in the general population [16]. Likewise, the positive association of baseline fasting insulin, HOMA-IR, and insulin-glucose ratio (IGR) with incident hypertension among an Iranian population has been previously reported [17].

It has been shown that beside baseline values, changes in classic risk factors including body weight, waist circumference (WC), and serum uric acid concentration play important roles in the development of hypertension [18–20]. To the best of our knowledge, there is no data regarding the impact of changes in fasting insulin and IR indices on incident hypertension. Considering the high incidence of both IR and hypertension among Iranian population [21], in the current study we aimed to examine the association of changes in levels of fasting insulin and IR indices including HOMA-IR and IGR over approximately 3 years with incident hypertension in an adult Middle-Eastern population.

## Methods

### Study design and population

The Tehran Lipid and Glucose Study (TLGS) is a large long term community-based prospective study, being conducted on a representative sample of residents from district No. 13 of Tehran, the capital city of Iran. The TLGS consists of two components: First, a baseline cross-sectional study (1999–2001), in which a representative population, aged over 3 years, participated. Second, a prospective study still in progress; detailed descriptions of the study have been published elsewhere [22]. Based on the TLGS protocol, the whole population was followed at approximately 3-year intervals; until Oct 2017, five follow-up examinations were accomplished for all participants [(2002–2005), (2005–2008), (2008–2011), (2011–2014), and (2014–2017)].

**Fig. 1:**
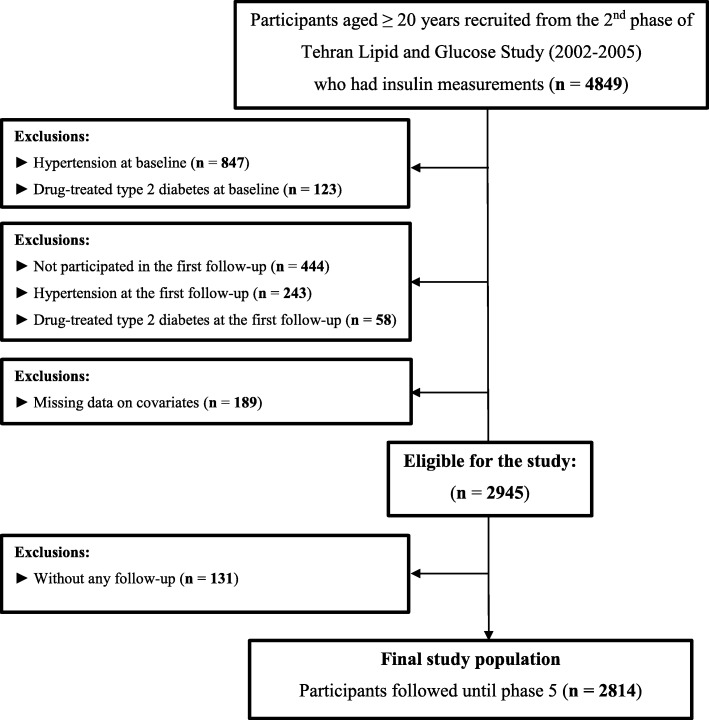
Flowchart of the study population

Due to the need to update physical activity questionnaire, we used the data from participants in the second phase of TLGS who had insulin data (2002–2005); hence, there were 4 follow-ups for participants who enrolled in the study. In the current study, we enrolled 4849 individuals aged ≥20 years, who were evaluated for the effects of changes in fasting insulin and IR indices between baseline (2002–2005) and the first follow-up (2005–2008) on incident hypertension; of these, individuals with prevalent hypertension at baseline (*n* = 847) and the first examination (*n* = 243) were excluded. Moreover, considering the effects of some glucose-lowering medications on insulin levels and IR indices, those with drug-treated type 2 diabetes (T2D) at baseline (*n* = 123) or at the first follow-up (*n* = 58) were excluded. Of those involved in the baseline survey, 444 individuals who did not participate in the first follow-up visit and 189 participants with missing data on covariates at baseline or at the first follow-up were also excluded, leaving 2945 participants. Finally, after further exclusion of participants without any follow-up (*n* = 131), 2814 subjects (1123 men and 1691 women) were remained, who were monitored for a median period of 6.32 years after the first examination (Fig. 1).

### Clinical and laboratory measurements

Baseline information including demographic data, education, medications use, past medical history of cardiovascular diseases (CVD) and smoking behavior were collected by a trained interviewer, using a pretested questionnaire during private interviews. Weight was measured, while subjects were minimally clothed with shoes removed, using digital electronic weighing scale (Seca 707, Seca Corp., Hanover, MD, USA; range 0.1–150 kg) and recorded to the nearest 100 g. Height was measured in a standing position without shoes, using a tape meter with shoulders in normal alignment. BMI was calculated as weight in kilograms divided by square of height in meters. WC was measured at the level of umbilicus.

Blood pressure was measured twice using a standardized mercury sphygmomanometer (calibrated by Iranian Institute of Standards and Industrial Researches) on the right arm in sitting position after at least 15 min of rest; and the average of two measurements was considered as the participant’s blood pressure.

From each individual a venous blood sample was collected between 7:00 and 9:00 AM after 12–14 h overnight fasting, and centrifuged within 30–45 min of collection.

Fasting plasma glucose (FPG) was measured by an enzymatic colorimetric assay using the glucose oxidase method; both intra- and inter-assay coefficients of variation (CVs) at baseline and follow-up surveys were < 2.3%. Serum creatinine was assayed using the photometric Jaffe method. Serum total cholesterol (TC) and triglycerides (TG) were measured using enzymatic calorimetric method with cholesterol esterase and cholesterol oxidase, and glycerol phosphate oxidase, respectively. High-density lipoprotein cholesterol (HDL-C) was assayed after precipitation of the apo-lipoprotein B containing lipoproteins with phosphotungistic acid. Both intra- and inter-assay CVs were below 2.1 and 3.0%, respectively, for TC, TG, and HDL-C, in all baseline and follow-up assays. All biochemical analyses, except insulin, were performed at the TLGS research laboratory on the day of blood collection using commercial kits (Pars Azmoon Inc., Tehran, Iran) and a Selectra 2 chemistry auto-analyzer (Vital Scientific, Spankeren, The Netherlands).

Aliquots of serum samples were stored at − 70 °C and transferred to the hormone laboratory of Research Institute for Endocrine Sciences for insulin assay. Fasting insulin was measured by the electrochemiluminescence immunoassay method using Roche Diagnostics kits and Roche/Hitachi Cobas e-411 analyzer (GmbH, Mannheim, Germany). Lyophilized quality control material (Lyphochek Immunoassay Plus Control; Bio-Rad Laboratories, Irvine, CA, USA) was used to monitor precision of assays. Intra- and inter-assay CVs were 1.2 and 3.5%, respectively. HOMA-IR was calculated as [FPG (mmol/L) × fasting insulin (mU/L)/22.5] [6]. IGR was calculated as [fasting insulin (mU/L)/FPG (mg/dL)].

The Chronic Kidney Disease Epidemiology Collaboration (CKD-EPI) creatinine equation was used to calculate estimated glomerular filtration rate (eGFR). As a single equation, CKD-EPI is expressed as follows:

eGFR = 141 × min (S_Cr_/κ, 1)^α^ × max (S_Cr_/κ, 1)^−1.209^ × 0.993^Age^ × 1.018 [if female] × 1.159 [if Black]

In this equation, S_cr_ is serum Cr in mg/dL; κ is 0.7 and 0.9 for women and men, respectively, and α is − 0.329 and − 0.411 for men and women, respectively. Min indicates the minimum of S_cr_/κ or 1, and max indicates maximum of S_cr_/κ or 1 [23].

### Definition of terms

In the present study, history of CVD was obtained by asking participants whether they had experienced any coronary heart disease (stable or unstable angina, or myocardial infarction), or coronary angiography, or cerebrovascular occurrences. A current smoker was defined as a person who smokes cigarettes daily or occasionally. Individuals were divided into three categories according to self-reported education status: < 6 years, 6–12 years, and ≥ 12 years.

Modifiable Activity Questionnaire (MAQ) that measured all three types of activity including leisure time, job, and household activities in the past year was used to evaluate physical activity levels [24]. Participants who had achieved a minimum of at least 600 MET (metabolic equivalent task)-minutes per week were considered as physically active.

Hypertension at baseline and during follow-up visits was defined as systolic blood pressure (SBP) ≥ 140 mmHg or diastolic blood pressure (DBP) ≥ 90 mmHg, or taking anti-hypertensive medications.

### Statistical analysis

To show baseline characteristics of participants, continuous variables are expressed as the mean ± standard deviation (SD) or median (interquartile range) for normal and skewed distributed variables, respectively, and frequency (%) for categorical variables. Baseline characteristics according to quartiles of changes in fasting insulin and IR indices were compared using ANOVA, Kruskal-Wallis, and Chi-square tests as appropriate.

The incidence rate of hypertension and 95% confidence interval (CI) were calculated by dividing the total number of incident cases to the sum of person-times of follow-up.

Event date for incident cases of hypertension was defined as mid-time between the date of follow-up visit at which hypertension was detected for the first time and the most recent follow-up visit preceding the diagnosis; the follow-up time was drawn from the difference between the calculated mid-time date and the date at which the subjects entered the study. For censored participants, survival time was calculated as the interval between the first and the last observation dates. Follow-up duration and incidence rates were calculated using the measured survival time.

Changes in fasting insulin levels and IR indices between baseline and the first follow-up were calculated to evaluate their impacts on incident hypertension; we categorized the exact amount of changes for insulin levels and IR indices into quartiles. The interaction of quartiles of changes in insulin, HOMA-IR, and IGR with gender was examined in fully-adjusted models using the log–likelihood ratio test. Since no interaction was found, the main analyses were performed on whole population to reach full statistical power.

Cox proportional hazard regression was used to assess the associations of quartiles of insulin, HOMA-IR, and IGR changes with incident hypertension, considering 1st quartile as reference, in whole population. Moreover, we examined the trends of quartiles for changes in insulin and IR indices in different models including: model 1, adjusted for age, sex, and baseline levels of insulin, HOMA-IR, or IGR as appropriate for corresponding model; model 2, further adjusted for smoking, physical activity, educational level, marital status, history of CVD, and baseline levels of SBP, DBP, BMI, eGFR, TG, TC, HDL-C, and FPG (only for insulin change); model 3, further adjusted for BMI change. The proportional hazard assumption in the Cox models was assessed with the Schoenfild residual test; all proportionality assumptions were appropriate.

We assessed Akaike’s information criteria (AIC) (a statistical estimate of the trade-off between the likelihood of a model against its complexity) as indicator for goodness of fit of each predictive model. A lower value of AIC indicates a better model fit. The discrimination ability of each model was calculated using the Harrell’s C statistic; it ranges from 0.0 to 1.0 and a C-index equal to 1.0 indicates perfect discrimination [25].

Statistical analyses were performed using SPSS for windows version 20 and STATA version 12 and *P*-value below 0.05 was considered statistically significant.

## Results

Overall, data of 2814 (men = 1123) individuals with a mean (SD) age of 39.4 (13.0) years were included in the analyses. Table 1 illustrates the baseline characteristics of study participants by quartiles of HOMA-IR changes. Accordingly, there were significant differences for age, sex, BMI, WC, and serum FPG, TC, TG levels, and incident hypertension among quartiles of HOMA-IR changes. Furthermore, Additional files 1 and 2: Tables S1 and S2 reveal the baseline characteristics by quartiles of insulin and IGR changes, respectively. Changing values of anthropometrics, SBP, DBP, and FPG over 3 years have been summarized in Table 2, by quartiles of HOMA-IR changes and in Additional files 3 and 4: Tables S3 and S4, according to quartiles of insulin and IGR changes, as well. Accordingly, we observed generally increasing trends for changes in BMI, WC, SBP, DBP, and FPG levels from 1st to 4th quartiles of fasting insulin and IR indices’ changes in all participants.

**Table 1:** Baseline characteristics of the study population by quartiles of HOMA-IR changes

	Quartiles of HOMA-IR changes					
	1st	2nd	3rd	4th	Total	
	(< − 0.550)	(≥ − 0.550 – < − 0.056)	(≥ − 0.056 – < 0.416)	(≥ 0.416)		
	(*n* = 703)	(*n* = 704)	(*n* = 704)	(n = 703)	(*n* = 2814)	*P* value^a^
Male gender, n (%)	243 (34.6)	296 (42.0)	288 (40.9)	296 (42.1)	1123 (39.9)	0.010
Age, years	37.5 (12.6)	40.1 (13.1)	40.6 (13.6)	39.5 (12.4)	39.4 (13.0)	< 0.001
BMI, Kg/m^2^	27.3 (4.6)	26.4 (4.3)	26.2 (4.6)	26.9 (4.5)	26.7 (4.5)	< 0.001
WC, cm	89.6 (12.2)	87.8 (11.5)	87.2 (11.5)	89.5 (11.7)	88.5 (11.8)	< 0.001
Education level, n (%)						0.235
<6 years	129 (18.3)	141 (20.0)	164 (23.3)	154 (21.9)	588 (20.9)	
6–12 years	445 (63.3)	432 (61.4)	423 (60.1)	440 (62.6)	1740 (61.8)	
≥ 12 years	129 (18.3)	131 (18.6)	117 (16.6)	109 (15.5)	486 (17.3)	
Marital status, n (%)						0.666
Married	549 (78.1)	545 (77.4)	563 (80.0)	538 (76.5)	2195 (78.0)	
Divorced/widowed	26 (3.7)	33 (4.7)	30 (4.3)	36 (5.1)	125 (4.4)	
Single	128 (18.2)	126 (17.9)	111 (15.8)	129 (18.3)	494 (17.6)	
Physical activity, n (%)						0.638
<600	255 (36.3)	252 (35.8)	247 (35.1)	269 (38.3)	1023 (36.4)	
≥ 600	448 (63.7)	452 (64.2)	457 (64.9)	434 (61.7)	1791 (63.6)	
SBP, mmHg	109.7 (11.1)	109.7 (11.1)	109.1 (12.0)	110.0 (11.5)	109.6 (11.4)	0.580
DBP, mmHg	72.1 (8.1)	71.5 (8.1)	71.1 (8.4)	72.0 (8.2)	71.7 (8.2)	0.079
Smoker, n (%)						0.149
Never/past	635 (90.3)	613 (87.1)	630 (89.5)	614 (87.3)	2492 (88.6)	
Current	68 (9.7)	91 (12.9)	74 (10.5)	89 (12.7)	322 (11.4)	
History of CVD, n (%)	7 (1.0)	6 (0.9)	6 (0.9)	11 (1.6)	30 (1.1)	0.512
eGFR, mL/min/1.73 m^2^	78.8 (13.1)	78.2 (13.4)	78.3 (13.1)	79.0 (13.0)	78.6 (13.1)	0.551
FPG, mmol/L	5.00 (4.77–5.38)	4.94 (4.66–5.16)	4.83 (4.55–5.11)	4.83 (4.55–5.16)	4.88 (4.61–5.22)	< 0.001
TC, mmol/L	4.90 (1.00)	4.83 (1.01)	4.75 (0.95)	4.86 (1.07)	4.84 (1.01)	0.040
TG, mmol/L	1.45 (1.03–2.15)	1.32 (0.94–1.88)	1.27 (0.94–1.81)	1.41 (0.94–2.08)	1.36 (0.96–1.98)	< 0.001
HDL-C, mmol/L	1.01 (0.26)	1.03 (0.28)	1.03 (0.27)	1.00 (0.26)	1.02 (0.27)	0.066
Incident hypertension, n (%)	142 (20.2)	129 (18.3)	142 (20.2)	181 (25.7)	594 (21.1)	0.004

Data are shown as mean (standard deviation), median (interquartile range), or number (proportion) as appropriate

*HOMA-IR* homeostasis model assessment of insulin resistance, *BMI* body mass index, *WC* waist circumference, *SBP* systolic blood pressure, *DBP* diastolic blood pressure, *CVD* cardiovascular disease, *eGFR* estimated glomerular filtration rate, *FPG* fasting plasma glucose, *TC* total cholesterol, *TG* triglycerides, *HDL-C* high density lipoprotein cholesterol

^a^ *P* values for difference across all quartiles of HOMA-IR changes were calculated with ANOVA, Kruskal-Wallis, and Chi-square tests, as appropriate

**Table 2:** Three-year changes in anthropometric, blood pressures, and fasting plasma glucose by quartiles of HOMA-IR changes

	Quartiles of HOMA-IR changes					
	1st	2nd	3rd	4th	Total	*P* value^a^
	(< − 0.550)	(≥ − 0.550 – < − 0.056)	(≥ − 0.056 – < 0.416)	(≥ 0.416)		
	(*n* = 703)	(*n* = 704)	(*n* = 704)	(*n* = 703)	(*n* = 2814)	
BMI, Kg/m^2^	− 0.25 (1.91)	0.31 (1.64)	0.49 (2.17)	1.07 (1.90)	0.40 (1.97)	< 0.001
WC, cm	− 1.17 (6.93)	0.57 (6.25)	1.25 (6.35)	2.63 (6.78)	0.82 (6.72)	< 0.001
SBP, mmHg	− 2.49 (10.76)	− 2.30 (10.94)	− 0.07 (11.36)	0.29 (11.03)	− 1.14 (11.09)	< 0.001
DBP, mmHg	− 1.70 (8.69)	− 1.15 (8.39)	− 0.73 (8.68)	0.36 (8.74)	− 0.80 (8.65)	< 0.001
FPG, mmol/L	− 0.26 (0.69)	− 0.13 (0.44)	0.07 (0.39)	0.31 (0.80)	− 0.002 (0.64)	< 0.001

*HOMA-IR* homeostasis model assessment of insulin resistance, *BMI* body mass index, *WC* waist circumference, *SBP* systolic blood pressure, *DBP* diastolic blood pressure, *FPG* fasting plasma glucose

Data are shown as mean (standard deviation)

^a^ *P* values for difference across all quartiles of HOMA-IR changes were calculated with ANOVA test

During a median follow-up of 6.32 years (interquartile range: 5.76–6.84) after the first examination, 594 participants experienced hypertension, including 253 men and 341 women; corresponding incidence rates per 10,000 person-years (95% CI) were 413.3 (365.7–467.2) and 357.2 (321.3–397.2), respectively.

Results of the Cox regression models, investigating the relation between 3-year changes of fasting insulin, HOMA-IR, and IGR with subsequent hypertension risk are presented in Table 3. Accordingly, changes in levels of fasting insulin and IR indices showed significant increasing trends for incident hypertension, moving from 1st quartile to 4th quartile. Specifically, compared to reference, the 4th quartile of insulin, HOMA-IR and IGR changes showed significant higher risk for incident hypertension in different models, excluding the 4th quartile of HOMA-IR changes in model 3 [hazard ratio: 1.18 (0.92–1.52)]. Focusing on model fitness as assessed by AIC, we did not find superiority for changes in fasting insulin or each of IR indices to predict incident hypertension. Moreover, the discriminatory powers of changes in fasting insulin and IR indices as assessed by C index were similar (i.e. about 80%).

**Table 3:** Multivariable-adjusted hazard ratios of incident hypertension by quartiles of changes in fasting serum insulin, HOMA-IR, and IGR in whole population

	HR (95% CI)				*P* for trend	AIC	C index %
	1st (reference)	2nd	3rd	4th			
Insulin^a^							
Model 1	1.00	1.15 (0.88–1.50)	1.48 (1.14–1.92)	1.85 (1.44–2.37)	< 0.001	8920.4	71.6
Model 2	1.00	1.02 (0.78–1.33)	1.37 (1.05–1.77)	1.48 (1.15–1.90)	0.001	8572.3	79.7
Model 3	1.00	0.96 (0.73–1.25)	1.27 (0.98–1.66)	1.31 (1.01–1.69)	0.015	8556.7	80.1
HOMA-IR^b^							
Model 1	1.00	0.99 (0.76–1.28)	1.15 (0.89–1.49)	1.65 (1.30–2.10)	< 0.001	8925.9	71.4
Model 2	1.00	0.86 (0.66–1.12)	1.08 (0.83–1.40)	1.34 (1.05–1.71)	0.002	8572.0	79.6
Model 3	1.00	0.81 (0.63–1.06)	1.00 (0.77–1.30)	1.18 (0.92–1.52)	0.016	8555.6	80.1
IGR^c^							
Model 1	1.00	1.30 (1.00–1.69)	1.62 (1.25–2.09)	1.98 (1.54–2.54)	< 0.001	8925.0	71.6
Model 2	1.00	1.28 (0.99–1.67)	1.52 (1.17–1.97)	1.68 (1.31–2.17)	< 0.001	8568.1	79.8
Model 3	1.00	1.21 (0.93–1.58)	1.40 (1.08–1.82)	1.53 (1.18–1.98)	0.008	8553.8	80.2

*HOMA-IR* homeostasis model assessment of insulin resistance, *IGR* insulin-glucose ratio, *HR* hazard ratio, *CI* confidence interval, *AIC* Akaike’s information criteria, *CVD* cardiovascular disease, *SBP* systolic blood pressure, *DBP* diastolic blood pressure, *BMI* body mass index, *FPG* fasting plasma glucose, *TC* total cholesterol, *TG* triglycerides, *HDL-C* high density lipoprotein cholesterol, *eGFR* estimated glomerular filtration rate

^a^Model 1: adjusted for age, sex, and baseline insulin; Model 2: model 1 + smoking, physical activity, marital status, history of CVD, education level, and baseline levels of SBP, DBP, BMI, FPG, TC, TG, HDL-C, and eGFR; Model 3: model 2 + BMI changes

^b^Model 1: adjusted for age, sex, and baseline HOMA-IR; Model 2: model 1 + smoking, physical activity, marital status, history of CVD, education level, and baseline levels of SBP, DBP, BMI, TC, TG, HDL-C, and eGFR; Model 3: model 2 + BMI changes

^c^Model 1: adjusted for age, sex, and baseline IGR; Model 2: model 1 + smoking, physical activity, marital status, history of CVD, education level, and baseline levels of SBP, DBP, BMI, TC, TG, HDL-C, and eGFR; Model 3: model 2 + BMI changes

We performed two sensitivity analyses to show robustness of findings. First, although no interaction was found between gender and quartiles of changes in fasting insulin (all *P*-values > 0.41), HOMA-IR (all *P*-values > 0.25), or IGR (all *P*-values > 0.15) in fully-adjusted models, the data analyses in each gender were presented in Additional files 5 and 6: Tables S5 and S6. Accordingly, the results among men were generally similar to those reported for the whole population; however, among women, the significant trends for quartiles of changes in fasting insulin and IR indices were found only in model 1. Second, although no interactions was found between baseline insulin level and quartiles of its changes (all *P*-values for interaction > 0.25) or HOMA-IR and quartiles of its changes (all *P*-values for interaction > 0.42), we reran the same analyses on participants with normal insulin or HOMA-IR values, using reference values of Iranian adults [26]. As shown in Additional file 7: Table S7, changes in fasting insulin levels increased risk of incident hypertension among those with normal insulin at baseline in different models.

## Discussion

This is the first study to have examined the impact of changes in fasting insulin and IR indices over approximately 3 years for incident hypertension in a population-based cohort. We found that over 6 years follow-up, there were significant trends in association between quartiles of changes in fasting insulin, HOMA-IR and IGR levels and incident hypertension, independent of important baseline confounders and BMI changes. Importantly, focusing on model fitness (as assessed by AIC) and discriminator power (as examined by C index), we did not find any superiority between these measures. Although we did not find any effect modification for gender on correlation of changes in fasting insulin levels or IR indices with incident hypertension, these associations were more prominent among men.

According to our data analysis, we found incidence rates of 413.3 and 357.2 per 10,000 person-years in men and women, respectively; slightly higher than those previously reported by Talaei et al. in the Isfahan Cohort Study which included both urban and rural areas [27]. The higher incidence rates in our study may be attributed to environmental impacts of urbanization on incident hypertension including different diet, lower physical activity, and exposure to ambient air pollution and traffic noise, especially in the metropolitan city of Tehran [28, 29]. Hence the incidence rates reported by Talaei et al. may have been diluted by pooling data of urban and rural areas.

Several previous studies have investigated the associations of insulin levels and IR indices, using snapshot values of these measures alone, with consequent hypertension [7–15, 17, 30, 31]. Sung et al., in a Korean study, showed that the highest quartiles of insulin and HOMA-IR were associated with 50 and 70% increased risk of incident hypertension, respectively [13]. Researchers from the community-based Multi-Ethnic Study of Atherosclerosis suggested that higher concentrations of insulin may contribute to the development of hypertension, in part through kidney disease and arterial stiffness [9]. Similarly, He et al. proposed that higher plasma insulin levels were associated with increased risk of hypertension in both African Americans and whites [8]. Furthermore, Saad et al. showed that the associations between hyperinsulinemia, IR, and blood pressure differ among racial groups and may be mediated by certain mechanisms active in whites, but not in Pima Indians or blacks [12]. Similar to these studies, we also previously reported the association between IR indices and incident hypertension among Iranian population [17]. A meta-analysis from 10 prospective studies showed that compared to the lowest quartile of fasting insulin concentrations, the highest quartile was associated with a pooled relative risk (95% CI) of 1.63 (1.35–1.97) for hypertension [30]. Another meta-analysis by Wang et al. that included prospective observational studies, showed that beside fasting insulin level, IR as estimated by HOMA index was also independently associated with an exacerbated risk of hypertension in the general population [16].

To the best of our knowledge, only one study conducted by Park et al. among 11,123 adults, examined the association between serum insulin changes for incident hypertension and showed that changing levels of fasting insulin appeared to be an independent determinant for future hypertension during a 4-year follow-up among non-diabetic healthy adults [11]. However, the second insulin measurement in Park et al. study was performed simultaneously with outcome assessment i.e. exposure did not precede hypertension; hence, it might not possible to talk exactly about cause and effect relationship. The current study conducted among Iranian adults adds to previous studies by showing that changes in serum insulin, HOMA-IR, and IGR levels precede the development of hypertension and might play a role in the pathogenesis of hypertension. Importantly, although a well-known association was reported between weight change and SBP levels [32], the significant associations of changes in insulin and IR indices with incident hypertension were sustained, independent of the BMI changes in this study. The mechanism remains unclear, but our results suggest that changes in fasting insulin and IR indices might play direct roles in increasing blood pressure through other mechanisms that do not completely capture by weight gain. Moreover, our results indirectly indicate that, if hyperinsulinemia could be corrected with any intervention, such as pharmacotherapy with Thiazolidinedione group of glucose-lowering medications [33], then the development of hypertension might be reduced.

Several mechanisms have been suggested to explain the association between IR and hypertension incidence. First, hyperinsulinemia activates the sympathetic nervous system and renin-angiotensin pathway, resulting in SBP elevation through increasing catecholamine levels, a phenomenon that is independent of blood glucose [4, 34]. Second, elevated serum insulin levels decrease urinary sodium excretion by increasing distal sodium reabsorption, leading to sodium and fluid retention [35]. Third, hyperinsulinemia also might diminish endothelium-dependent vasodilation in large arteries via increased oxidative stress [36].

The main strength of this study lies on its large sample size and a long-term follow-up duration. To the best of our knowledge, previous longitudinal studies have discussed roles of both IR and compensatory hyperinsulinemia in the development of hypertension, using baseline values of these measures alone; thus, this is the first study extending previous studies by evaluating changes of fasting insulin, HOMA-IR, and IGR levels with risk of subsequent hypertension.

Limitations of the present study include using HOMA-IR as a good surrogate measure of IR in epidemiologic studies rather than using hyperinsulinemic euglycemic clamp as a gold standard test for assessment of IR [37]. Second, according to TLGS protocol, the data regarding alcohol intake is not assessed for participants and alcohol consumption was not included as a covariate in the analyses. Third, since the present study was conducted among Persian ethnicities resident in Tehran, as a Middle-Eastern population, generalizing the results to other populations should thus be done cautiously.

## Conclusions

We found that 3-year changes of fasting insulin and IR indices are strong risk factors for developing hypertension among normotensive healthy adults without known diabetes, independent of large set of covariates and BMI changes. Further prospective and interventional studies aiming to reduce hyperinsulinemia are needed to confirm the significant roles of hyperinsulinemia and IR on the development of hypertension.

## Supplementary information

**Additional file 1: Table S1.** Baseline characteristics of the study population by quartiles of insulin changes.

**Additional file 2: Table S2.** Baseline characteristics of the study population by quartiles of IGR changes.

**Additional file 3: Table S3.** Three-year changes in anthropometric, blood pressures, and fasting plasma glucose by quartiles of insulin changes.

**Additional file 4: Table S4.** Three-year changes in anthropometric, blood pressures, and fasting plasma glucose by quartiles of IGR changes.

**Additional file 5: Table S5.** Multivariable-adjusted hazard ratios of incident hypertension by quartiles of changes in fasting serum insulin, HOMA-IR, and IGR among men.

**Additional file 6: Table S6.** Multivariable-adjusted hazard ratios of incident hypertension by quartiles of changes in fasting serum insulin, HOMA-IR, and IGR among women.

**Additional file 7: Table S7.** Multivariable-adjusted hazard ratios of incident hypertension by quartiles of changes in fasting serum insulin and HOMA-IR among participants with normal insulin or HOMA-IR levels at baseline.

## Data Availability

Study data has been extracted from the Tehran Lipid and Glucose study (TLGS), a longitudinal and population-based prospective study performed on a representative sample of an urban population of Tehran (the capital of Iran). The statistical analyses/codes for the current study can be made available from the corresponding author on reasonable request.

## Abbreviations

AIC: Akaike’s information criteria
BMI: Body mass index
CI: Confidence interval
CKD-EPI: Chronic kidney disease Epidemiology Collaboration
CV: Coefficient of variation
CVD: Cardiovascular diseases
DBP: Diastolic blood pressure
eGFR: Estimated glomerular filtration rate
FPG: Fasting plasma glucose
HDL-C: High-density lipoprotein cholesterol
HOMA-IR: Homeostasis model assessment of insulin resistance
HR: Hazard ratio
IGR: Insulin-glucose ratio
IR: Insulin resistance
MAQ: Modifiable Activity Questionnaire
MET: Metabolic equivalent task
SBP: Systolic blood pressure
TC: Total cholesterol
TG: Triglycerides
TLGS: Tehran Lipid and Glucose Study
WC: Waist circumference
