# Supplementary material for: Impact of 3-year changes in fasting insulin and insulin resistance indices on incident hypertension: Tehran lipid and glucose study
Source: Nutr Metab (Lond). 2019 Nov 9;16:76. doi: 10.1186/s12986-019-0402-3 (PMC6842481; doi:10.1186/s12986-019-0402-3)
Supplement: Supplementary file 1 — Additional file 1: Table S1. Baseline characteristics of the study population by quartiles of insulin changes. [file 12986_2019_402_MOESM1_ESM.docx]

| **Supplementary Table 1** Baseline characteristics of the study population by quartiles of insulin changes | | | | | | |  |
| --- | --- | --- | --- | --- | --- | --- | --- |
|  | Quartiles of insulin changes | | | |  | |  |
|  | 1^st^ | 2^nd^ | 3^rd^ | 4^th^ | |  | |
|  | (< -2.350) | (≥ -2.350 – < -0.255) | (≥ -0.255 – < 1.803) | (≥ 1.803) | | *P* value^a^ | |
|  | (n = 703) | (n = 704) | (n = 704) | (n = 703) | |  | |
| Male gender, n (%) | 233 (33.1) | 300 (42.6) | 294 (41.8) | 296 (42.1) | | < 0.001 | |
| Age, years | 36.9 (12.6) | 41.2 (13.2) | 40.3 (13.3) | 39.3 (12.4) | | < 0.001 | |
| BMI, Kg/m^2^ | 27.1 (4.6) | 26.3 (4.3) | 26.4 (4.6) | 26.8 (4.5) | | 0.002 | |
| WC, cm | 89.2 (12.3) | 87.9 (11.3) | 87.9 (11.8) | 89.2 (11.7) | | 0.037 | |
| Education level, n (%) |  |  |  |  | | 0.099 | |
| ˂6 years | 122 (17.4) | 155 (22.0) | 163 (23.2) | 148 (21.1) | |  | |
| 6-12 years | 454 (64.6) | 418 (59.4) | 423 (60.1) | 445 (63.3) | |  | |
| ≥12 years | 127 (18.1) | 131 (18.6) | 118 (16.8) | 110 (15.6) | |  | |
| Marital status, n (%) |  |  |  |  | | 0.256 | |
| Married | 542 (77.1) | 565 (80.3) | 553 (78.6) | 535 (76.1) | |  | |
| Divorced/widowed | 26 (3.7) | 34 (4.8) | 28 (4.0) | 37 (5.3) | |  | |
| Single | 135 (19.2) | 105 (14.9) | 123(17.5) | 131 (18.6) | |  | |
| Physical activity, n (%) |  |  |  |  | | 0.490 | |
| ˂ 600 | 261 (37.1) | 242 (34.4) | 252 (35.8) | 268 (38.1) | |  | |
| ≥ 600 | 442 (62.9) | 462 (65.6) | 452 (64.2) | 435 (61.9) | |  | |
| SBP, mmHg | 109.2 (10.8) | 110.4 (11.3) | 109.1 (11.7) | 109.7 (11.7) | | 0.123 | |
| DBP, mmHg | 72.0 (8.2) | 71.6 (8.0) | 71.2 (8.4) | 71.9 (8.3) | | 0.242 | |
| Smoker, n (%) |  |  |  |  | | 0.231 | |
| Never/past | 634 (90.2) | 629 (89.3) | 615 (87.4) | 614 (87.3) | |  | |
| Current | 69 (9.8) | 75 (10.7) | 89 (12.6) | 89 (12.7) | |  | |
| History of CVD, n (%) | 8 (1.1) | 3 (0.4) | 8 (1.1) | 11 (1.6) | | 0.216 | |
| eGFR, mL/min/1.73 m^2^ | 79.1 (13.3) | 77.5 (13.1) | 79.0 (13.3) | 78.7 (12.8) | | 0.083 | |
| FPG, mmol/L | 4.94 (4.72-5.27) | 4.94 (4.66-5.22) | 4.88 (4.61-5.16) | 4.83 (4.55-5.16) | | < 0.001 | |
| TC, mmol/L | 4.87 (0.99) | 4.82 (1.02) | 4.80 (0.99) | 4.85 (1.04) | | 0.626 | |
| TG, mmol/L | 1.44 (1.01-2.09) | 1.34 (0.95-1.95) | 1.28 (0.94-1.82) | 1.38 (0.94-2.00) | | 0.004 | |
| HDL-C, mmol/L | 1.01 (0.27) | 1.04 (0.28) | 1.02 (0.26) | 1.00 (0.27) | | 0.107 | |
| Incident hypertension, n (%) | 129 (18.3) | 135 (19.2) | 155 (22.0) | 175 (24.9) | | 0.011 | |
| ^a^ *P* values for difference across all quartiles of insulin changes were calculated with ANOVA, Kruskal-Wallis, and Chi-square tests, as appropriate  Data are shown as mean (standard deviation), median (interquartile range), or number (proportion) as appropriate  *BMI* body mass index, *WC* waist circumference, *SBP* systolic blood pressure, *DBP* diastolic blood pressure, *CVD* cardiovascular disease, *eGFR* estimated glomerular filtration rate, *FPG* fasting plasma glucose, *TC* total cholesterol, *TG* triglycerides, *HDL-C* high density lipoprotein cholesterol | | | | | | |  |
