# Supplementary material for: Impact of 3-year changes in fasting insulin and insulin resistance indices on incident hypertension: Tehran lipid and glucose study
Source: Nutr Metab (Lond). 2019 Nov 9;16:76. doi: 10.1186/s12986-019-0402-3 (PMC6842481; doi:10.1186/s12986-019-0402-3)
Supplement: Supplementary file 2 — Additional file 2: Table S2. Baseline characteristics of the study population by quartiles of IGR changes. [file 12986_2019_402_MOESM2_ESM.docx]

| **Supplementary Table 2** Baseline characteristics of the study population by quartiles of IGR changes | | | | | |
| --- | --- | --- | --- | --- | --- |
|  | Quartiles of IGR changes | | | |  |
|  | 1^st^ | 2^nd^ | 3^rd^ | 4^th^ |  |
|  | (< -0.450) | (≥ -0.450 – < -0.042) | (≥ -0.042 – < 0.360) | (≥ 0.360) | *P* value^a^ |
|  | (n = 703) | (n = 704) | (n = 704) | (n = 703) |  |
| Male gender, n (%) | 239 (34.0) | 285 (40.5) | 310 (44.0) | 289 (41.1) | 0.001 |
| Age, years | 37.1 (12.8) | 41.6 (13.1) | 40.6 (13.0) | 38.5 (12.4) | < 0.001 |
| BMI, Kg/m^2^ | 27.0 (4.7) | 26.5 (4.2) | 26.4 (4.4) | 26.9 (4.7) | 0.034 |
| WC, cm | 88.8 (12.4) | 88.3 (11.2) | 88.2 (11.7) | 89.0 (11.8) | 0.525 |
| Education level, n (%) |  |  |  |  | 0.026 |
| ˂6 years | 119 (16.9) | 173 (24.6) | 151 (21.4) | 145 (20.6) |  |
| 6-12 years | 449 (63.9) | 412 (58.5) | 433 (61.5) | 446 (63.4) |  |
| ≥12 years | 135 (19.2) | 119 (16.9) | 120 (17.0) | 112 (15.9) |  |
| Marital status, n (%) |  |  |  |  | 0.016 |
| Married | 534 (76.0) | 576 (81.8) | 561 (79.7) | 524 (74.5) |  |
| Divorced/widowed | 30 (4.3) | 31 (4.4) | 28 (4.0) | 36 (5.1) |  |
| Single | 139 (19.8) | 97 (13.8) | 115 (16.3) | 143 (20.3) |  |
| Physical activity, n (%) |  |  |  |  | 0.066 |
| ˂600 | 266 (37.8) | 227 (32.2) | 260 (36.9) | 270 (38.4) |  |
| ≥600 | 437 (62.2) | 477 (67.8) | 444 (63.1) | 433 (61.6) |  |
| SBP, mmHg | 109.3 (11.0) | 110.2 (11.2) | 109.6 (11.7) | 109.4 (11.6) | 0.524 |
| DBP, mmHg | 72.0 (8.1) | 71.8 (7.9) | 71.3 (8.5) | 71.6 (8.3) | 0.465 |
| Smoker, n (%) |  |  |  |  | 0.028 |
| Never/past | 633 (90.0) | 638 (90.6) | 608 (86.4) | 613 (87.2) |  |
| Current | 70 (10.0) | 66 (9.4) | 96 (13.6) | 90 (12.8) |  |
| History of CVD, n (%) | 7 (1.0) | 5 (0.7) | 7 (1.0) | 11 (1.6) | 0.463 |
| eGFR, mL/min/1.73 m^2^ | 79.4 (13.7) | 77.3 (12.7) | 78.9 (13.1) | 78.8 (12.9) | 0.019 |
| FPG, mmol/L | 4.88 (4.61-5.22) | 4.88 (4.61-5.27) | 4.94 (4.66-5.22) | 4.88 (4.61-5.16) | 0.009 |
| TC, mmol/L | 4.84 (0.99) | 4.85 (1.04) | 4.83 (0.96) | 4.84 (1.05) | 0.981 |
| TG, mmol/L | 1.41 (0.99-2.09) | 1.34 (0.96-1.97) | 1.30 (0.94-1.83) | 1.36 (0.94-1.99) | 0.016 |
| HDL-C, mmol/L | 1.01 (0.26) | 1.03 (0.28) | 1.02 (0.26) | 1.01 (0.27) | 0.361 |
| Incident hypertension, n (%) | 119 (16.9) | 145 (20.6) | 159 (22.6) | 171 (24.3) | 0.005 |
| ^a^ *P* values for difference across all quartiles of IGR changes were calculated with ANOVA, Kruskal-Wallis, and Chi-square tests, as appropriate  Data are shown as mean (standard deviation), median (interquartile range), or number (proportion) as appropriate  *IGR* insulin-glucose ratio, *BMI* body mass index, *WC* waist circumference, *SBP* systolic blood pressure, *DBP* diastolic blood pressure, *CVD* cardiovascular disease, *eGFR* estimated glomerular filtration rate, *FPG* fasting plasma glucose, *TC* total cholesterol, *TG* triglycerides, *HDL-C* high density lipoprotein cholesterol | | | | | |
