# Supplementary material for: Impact of 3-year changes in fasting insulin and insulin resistance indices on incident hypertension: Tehran lipid and glucose study
Source: Nutr Metab (Lond). 2019 Nov 9;16:76. doi: 10.1186/s12986-019-0402-3 (PMC6842481; doi:10.1186/s12986-019-0402-3)
Supplement: Supplementary file 3 — Additional file 3: Table S3. Three-year changes in anthropometric, blood pressures, and fasting plasma glucose by quartiles of insulin changes. [file 12986_2019_402_MOESM3_ESM.docx]

| **Supplementary Table 3** Three-year changes in anthropometric, blood pressures, and fasting plasma glucose by quartiles of insulin changes | | | | | |
| --- | --- | --- | --- | --- | --- |
|  | Quartiles of insulin changes | | | |  |
|  | 1^st^ | 2^nd^ | 3^rd^ | 4^th^ |  |
|  | (< -2.350) | (≥ -2.350 – < -0.255) | (≥ -0.255 – < 1.803) | (≥ 1.803) | *P* value^a^ |
|  | (n = 703) | (n = 704) | (n = 704) | (n = 703) |  |
| BMI, Kg/m^2^ | -0.21 (1.93) | 0.27 (1.78) | 0.48 (2.03) | 1.08 (1.92) | < 0.001 |
| WC, cm | -1.13 (7.10) | 0.53 (6.04) | 1.12 (6.44) | 2.77 (6.68) | < 0.001 |
| SBP, mmHg | -2.43 (10.62) | -2.31 (10.97) | -0.13 (11.58) | 0.30 (10.93) | < 0.001 |
| DBP, mmHg | -1.72 (8.59) | -1.18 (8.44) | -0.65 (8.57) | 0.34 (8.88) | < 0.001 |
| FPG, mmol/L | -0.12 (0.78) | -0.05 (0.59) | 0.01 (0.57) | 0.15 (0.56) | < 0.001 |
| ^a^ *P v*alues for difference across all quartiles of insulin changes were calculated with ANOVA test  Data are shown as mean (standard deviation)  *BMI* body mass index, *WC* waist circumference, *SBP* systolic blood pressure, *DBP* diastolic blood pressure, *FPG* fasting plasma glucose | | | | | |
