# Supplementary material for: Impact of 3-year changes in fasting insulin and insulin resistance indices on incident hypertension: Tehran lipid and glucose study
Source: Nutr Metab (Lond). 2019 Nov 9;16:76. doi: 10.1186/s12986-019-0402-3 (PMC6842481; doi:10.1186/s12986-019-0402-3)
Supplement: Supplementary file 4 — Additional file 4: Table S4. Three-year changes in anthropometric, blood pressures, and fasting plasma glucose by quartiles of IGR changes. [file 12986_2019_402_MOESM4_ESM.docx]

| **Supplementary Table 4** Three-year changes in anthropometric, blood pressures, and fasting plasma glucose by quartiles of IGR changes | | | | | |
| --- | --- | --- | --- | --- | --- |
|  | Quartiles of IGR changes | | | |  |
|  | 1^st^ | 2^nd^ | 3^rd^ | 4^th^ | *P* value^a^ |
|  | (< -0.450) | (≥ -0.450 – < -0.042) | (≥ -0.042 – < 0.360) | (≥ 0.360) |  |
|  | (n = 703) | (n = 704) | (n = 704) | (n = 703) |  |
| BMI, Kg/m^2^ | -0.16 (2.00) | 0.26 (1.69) | 0.57 (1.66) | 0.95 (2.30) | < 0.001 |
| WC, cm | -0.80 (7.16) | 0.24 (6.09) | 1.50 (6.37) | 2.34 (6.79) | < 0.001 |
| SBP, mmHg | -2.35 (10.87) | -1.75 (10.86) | -0.49 (11.15) | 0.02 (11.34) | < 0.001 |
| DBP, mmHg | -1.78 (8.60) | -1.15 (8.26) | -0.52 (8.62) | 0.24 (9.01) | < 0.001 |
| FPG, mmol/L | 0.01 (0.84) | 0.02 (0.65) | -0.04 (0.55) | 0.00 (0.47) | 0.171 |
| ^a^ *P* values for difference across all quartiles of IGR changes were calculated with ANOVA test  Data are shown as mean (standard deviation)  *IGR* insulin-glucose ratio, *BMI* body mass index, *WC* waist circumference, *SBP* systolic blood pressure, *DBP* diastolic blood pressure, *FPG* fasting plasma glucose | | | | | |
