# Supplementary material for: Impact of 3-year changes in fasting insulin and insulin resistance indices on incident hypertension: Tehran lipid and glucose study
Source: Nutr Metab (Lond). 2019 Nov 9;16:76. doi: 10.1186/s12986-019-0402-3 (PMC6842481; doi:10.1186/s12986-019-0402-3)
Supplement: Supplementary file 5 — Additional file 5: Table S5. Multivariable-adjusted hazard ratios of incident hypertension by quartiles of changes in fasting serum insulin, HOMA-IR, and IGR among men. [file 12986_2019_402_MOESM5_ESM.docx]

| **Supplementary Table 5** Multivariable-adjusted hazard ratios of incident hypertension by quartiles of changes in fasting serum insulin, HOMA-IR, and IGR among men | | | | | | | |
| --- | --- | --- | --- | --- | --- | --- | --- |
|  |  | **HR (95 % CI)** | |  | ***P* for trend** | **AIC** | **C index %** |
|  | 1^st^ (reference) | 2^nd^ | 3^rd^ | 4^th^ |  |  |  |
| **Insulin^a^** |  |  |  |  |  |  |  |
| Model 1 | 1.00 | 1.10 (0.74-1.65) | 1.34 (0.90-2.00) | 1.99 (1.38-2.90) | < 0.001 | 3357.9 | 68.3 |
| Model 2 | 1.00 | 1.00 (0.66-1.49) | 1.20 (0.81-1.77) | 1.71 (1.17-2.48) | 0.008 | 3263.5 | 76.9 |
| Model 3 | 1.00 | 0.98 (0.65-1.48) | 1.18 (0.79-1.76) | 1.67 (1.13-2.46) | 0.014 | 3265.3 | 76.9 |
| **HOMA-IR^b^** |  |  |  |  |  |  |  |
| Model 1 | 1.00 | 1.35 (0.91-2.01) | 1.25 (0.83-1.89) | 2.36 (1.62-3.44) | < 0.001 | 3349.4 | 69.2 |
| Model 2 | 1.00 | 1.12 (0.76-1.66) | 1.02 (0.68-1.53) | 1.87 (1.28-2.72) | 0.001 | 3257.1 | 76.9 |
| Model 3 | 1.00 | 1.12 (0.75-1.65) | 1.01 (0.67-1.52) | 1.84 (1.25-2.71) | 0.001 | 3259.0 | 76.9 |
| **IGR^c^** |  |  |  |  |  |  |  |
| Model 1 | 1.00 | 1.29 (0.85-1.95) | 1.51 (1.003-2.28) | 2.34 (1.60-3.40) | < 0.001 | 3356.0 | 68.4 |
| Model 2 | 1.00 | 1.22 (0.81-1.85) | 1.37 (0.91-2.05) | 2.04 (1.40-2.97) | 0.001 | 3257.2 | 76.9 |
| Model 3 | 1.00 | 1.21 (0.79-1.83) | 1.35 (0.90-2.03) | 2.01 (1.36-2.95) | 0.001 | 3259.1 | 77.0 |
| **^a^Model 1:** adjusted for age and baseline insulin; **Model 2:** model 1 + smoking, physical activity, marital status, history of CVD, education level, and baseline levels of SBP, DBP, BMI, FPG, TC, TG, HDL-C, and eGFR; **Model 3:** model 2 + BMI changes  **^b^Model 1:** adjusted for age and baseline HOMA-IR; **Model 2:** model 1 + smoking, physical activity, marital status, history of CVD, education level, and baseline levels of SBP, DBP, BMI, TC, TG, HDL-C, and eGFR; **Model 3:** model 2 + BMI changes  **^c^Model 1:** adjusted for age and baseline IGR; **Model 2:** model 1 + smoking, physical activity, marital status, history of CVD, education level, and baseline levels of SBP, DBP, BMI, TC, TG, HDL-C, and eGFR; **Model 3:** model 2 + BMI changes  *HOMA-IR* homeostasis model assessment of insulin resistance, *IGR* insulin-glucose ratio, *HR* hazard ratio, *CI* confidence interval, *AIC* Akaike's information criteria, *CVD* cardiovascular disease, *SBP* systolic blood pressure, *DBP* diastolic blood pressure, *BMI* body mass index, *FPG* fasting plasma glucose, *TC* total cholesterol, *TG* triglycerides, *HDL-C* high density lipoprotein cholesterol, *eGFR* estimated glomerular filtration rate | | | | | | | |
