# Supplementary material for: Impact of 3-year changes in fasting insulin and insulin resistance indices on incident hypertension: Tehran lipid and glucose study
Source: Nutr Metab (Lond). 2019 Nov 9;16:76. doi: 10.1186/s12986-019-0402-3 (PMC6842481; doi:10.1186/s12986-019-0402-3)
Supplement: Supplementary file 6 — Additional file 6: Table S6. Multivariable-adjusted hazard ratios of incident hypertension by quartiles of changes in fasting serum insulin, HOMA-IR, and IGR among women. [file 12986_2019_402_MOESM6_ESM.docx]

| **Supplementary Table 6** Multivariable-adjusted hazard ratios of incident hypertension by quartiles of changes in fasting serum insulin, HOMA-IR, and IGR among women | | | | | | | |
| --- | --- | --- | --- | --- | --- | --- | --- |
|  |  | **HR (95 % CI)** | |  | ***P* for trend** | **AIC** | **C index %** |
|  | 1^st^ (reference) | 2^nd^ | 3^rd^ | 4^th^ |  |  |  |
| **Insulin^a^** |  |  |  |  |  |  |  |
| Model 1 | 1.00 | 1.05 (0.74-1.48) | 1.35 (0.96-1.90) | 1.53 (1.09-2.13) | 0.013 | 4751.4 | 74.2 |
| Model 2 | 1.00 | 0.94 (0.66-1.34) | 1.30 (0.92-1.84) | 1.24 (0.88-1.74) | 0.112 | 4513.7 | 82.1 |
| Model 3 | 1.00 | 0.86 (0.60-1.22) | 1.17 (0.83-1.65) | 1.06 (0.75-1.49) | 0.262 | 4491.7 | 82.8 |
| **HOMA-IR^b^** |  |  |  |  |  |  |  |
| Model 1 | 1.00 | 1.06 (0.75-1.50) | 1.25 (0.88-1.76) | 1.52 (1.10-2.12) | 0.035 | 4754.3 | 73.8 |
| Model 2 | 1.00 | 0.90 (0.64-1.28) | 1.26 (0.89-1.78) | 1.27 (0.91-1.77) | 0.084 | 4511.3 | 81.9 |
| Model 3 | 1.00 | 0.83 (0.59-1.18) | 1.13 (0.79-1.60) | 1.08 (0.77-1.51) | 0.252 | 4490.1 | 82.7 |
| **IGR^c^** |  |  |  |  |  |  |  |
| Model 1 | 1.00 | 1.19 (0.85-1.68) | 1.53 (1.09-2.14) | 1.60 (1.14-2.25) | 0.017 | 4754.1 | 74.0 |
| Model 2 | 1.00 | 1.18 (0.84-1.68) | 1.48 (1.05-2.08) | 1.34 (0.95-1.89) | 0.127 | 4513.0 | 82.2 |
| Model 3 | 1.00 | 1.11 (0.78-1.57) | 1.33 (0.94-1.87) | 1.18 (0.84-1.66) | 0.394 | 4491.6 | 82.8 |
| **^a^Model 1:** adjusted for age and baseline insulin; **Model 2:** model 1 + smoking, physical activity, marital status, history of CVD, education level, and baseline levels of SBP, DBP, BMI, FPG, TC, TG, HDL-C, and eGFR; **Model 3:** model 2 + BMI changes  **^b^Model 1:** adjusted for age and baseline HOMA-IR; **Model 2:** model 1 + smoking, physical activity, marital status, history of CVD, education level, and baseline levels of SBP, DBP, BMI, TC, TG, HDL-C, and eGFR; **Model 3:** model 2 + BMI changes  **^c^Model 1:** adjusted for age and baseline IGR; **Model 2:** model 1 + smoking, physical activity, marital status, history of CVD, education level, and baseline levels of SBP, DBP, BMI, TC, TG, HDL-C, and eGFR; **Model 3:** model 2 + BMI changes  *HOMA-IR* homeostasis model assessment of insulin resistance, *IGR* insulin-glucose ratio, *HR* hazard ratio, *CI* confidence interval, *AIC* Akaike's information criteria, *CVD* cardiovascular disease, *SBP* systolic blood pressure, *DBP* diastolic blood pressure, *BMI* body mass index, *FPG* fasting plasma glucose, *TC* total cholesterol, *TG* triglycerides, *HDL-C* high density lipoprotein cholesterol, *eGFR* estimated glomerular filtration rate | | | | | | | |
