# Supplementary material for: Impact of 3-year changes in fasting insulin and insulin resistance indices on incident hypertension: Tehran lipid and glucose study
Source: Nutr Metab (Lond). 2019 Nov 9;16:76. doi: 10.1186/s12986-019-0402-3 (PMC6842481; doi:10.1186/s12986-019-0402-3)
Supplement: Supplementary file 7 — Additional file 7: Table S7. Multivariable-adjusted hazard ratios of incident hypertension by quartiles of changes in fasting serum insulin and HOMA-IR among participants with normal insulin or HOMA-IR levels at baseline. [file 12986_2019_402_MOESM7_ESM.docx]

| **Supplementary Table 7** Multivariable-adjusted hazard ratios of incident hypertension by quartiles of changes in fasting serum insulin and HOMA-IR among participants with normal insulin or HOMA-IR levels at baseline | | | | | |
| --- | --- | --- | --- | --- | --- |
|  |  | **HR (95 % CI)** | |  | ***P* for trend** |
|  | 1^st^ (reference) | 2^nd^ | 3^rd^ | 4^th^ |  |
| **Insulin^a^** |  |  |  |  |  |
| Model 1 | 1.00 | 0.98 (0.73-1.32) | 1.20 (0.90-1.60) | 1.52 (1.14-2.03) | 0.002 |
| Model 2 | 1.00 | 0.94 (0.70-1.27) | 1.24 (0.93-1.66) | 1.36 (1.02-1.82) | 0.013 |
| Model 3 | 1.00 | 0.90 (0.67-1.21) | 1.18 (0.88-1.58) | 1.23 (0.92-1.65) | 0.057 |
| **HOMA-IR^b^** |  |  |  |  |  |
| Model 1 | 1.00 | 1.31 (0.68-2.52) | 1.46 (0.76-2.80) | 2.23 (1.18-4.24) | 0.008 |
| Model 2 | 1.00 | 1.09 (0.56-2.12) | 1.20 (0.62-2.33) | 1.62 (0.84-3.11) | 0.149 |
| Model 3 | 1.00 | 1.04 (0.53-2.02) | 1.06 (0.54-2.06) | 1.34 (0.69-2.60) | 0.513 |
| **^a^** Participants with baseline insulin 2.11 to 12.49 are considered as insulin sensitive (n = 2361)  **Model 1:** adjusted for age and sex; **Model 2:** model 1 + smoking, physical activity, marital status, history of CVD, education level, and baseline levels of SBP, DBP, BMI, FPG, TC, TG, HDL-C, and eGFR; **Model 3:** model 2 + BMI changes  **^b^** Participants with baseline HOMA-IR 0.94 to 1.68 are considered as insulin sensitive (n = 1011)  **Model 1:** adjusted for age and sex; **Model 2:** model 1 + smoking, physical activity, marital status, history of CVD, education level, and baseline levels of SBP, DBP, BMI, TC, TG, HDL-C, and eGFR; **Model 3:** model 2 + BMI changes  *HOMA-IR* homeostasis model assessment of insulin resistance, *HR* hazard ratio, *CI* confidence interval, *CVD* cardiovascular disease, *SBP* systolic blood pressure, *DBP* diastolic blood pressure, *BMI* body mass index, *FPG* fasting plasma glucose, *TC* total cholesterol, *TG* triglycerides, *HDL-C* high density lipoprotein cholesterol, *eGFR* estimated glomerular filtration rate | | | | | |
